# From glacial refugia to hydrological microrefugia: Factors and processes driving the persistence of the climate relict tree *Zelkova sicula*

**DOI:** 10.1002/ece3.7253

**Published:** 2021-02-25

**Authors:** Giuseppe Garfì, Francesco Carimi, Laurence Fazan, Alessandro Silvestre Gristina, Gregor Kozlowski, Salvatore Livreri Console, Antonio Motisi, Salvatore Pasta

**Affiliations:** ^1^ Institute of Biosciences and BioResources – CNR Palermo Italy; ^2^ Department of Biology and Botanical Garden University of Fribourg Fribourg Switzerland; ^3^ Natural History Museum Fribourg Fribourg Switzerland; ^4^ Shanghai Chenshan Plant Science Research Center Chinese Academy of Sciences Shanghai Chenshan Botanical Garden Songjiang China; ^5^ Marine Protected Area “Isole Egadi” Favignana Italy

**Keywords:** Cenozoic relicts, ecological plasticity, marginal habitats, rear edge populations, soil moisture, topographic attribute analyses

## Abstract

With only two tiny populations, the climate relict *Zelkova sicula* (Sicily, Italy) is one of the rarest trees in the world. It also represents the most marginal member of genus *Zelkova* that was widespread in the broadleaved forests thriving in warm–temperate climates throughout Eurasia until the Last Glacial Age. Occurring at the westernmost range of the genus under typical Mediterranean climate, the micro‐topographic settings have always appeared crucial for the survival of this relict. However, the factors and processes actually involved in its persistence in the current refugia, as well as the response of similar relict trees in arid environments, are poorly understood worldwide. In the aim to elucidate these aspects, in the two sites hosting *Z. sicula* analyses of topographical attributes were combined with investigations on soil moisture dynamics. Additionally, plants’ growth and spatial distribution patterns were analyzed to detect fine‐scale differences between populations and assess the possible ecological amplitude of the species. Results revealed that convergent topographies are basic determinants of microrefugia in arid environments. Within the investigated sites, underground moisture never decreases below 25%, buffering seasonal rainfall fluctuations. Therefore, hydrological microrefugia play a key role in decoupling from regional climate, supporting the target species in coping with an unsuitable climatic envelope. Additionally, the inter‐population variability of biometric attributes showed that individual growth is site‐dependent and the species retains a relative ecological plasticity, whereas the strongly clumped spatial patterns confirmed the common clonal growth. On one hand, deeply incised landforms have acted as effective hydrologic microrefugia, on the other clonality coupled with triploidy supposedly improved the resistance of *Z. sicula* to harsh environments, though entailing inability to reproduce sexually. Most likely, sterility and environmental/physical barriers that have existed for millennia have prevented this relict from leaving the last suitable microrefugia, resulting in the two current rear edge populations.

## Graphical abstract

Deeply incised landforms support microhabitats that are significantly moister than the surrounding matrix. They play a key role in decoupling from regional climate, supporting the climate relict tree *Zelkova sicula* in coping with an unsuitable climatic envelope and acting as effective hydrologic microrefugia. Clonality coupled with triploidy supposedly improved the resistance of this relict to harsh environments, but entailed inability to reproduce sexually. Sterility and environmental/physical barriers probably hamper since millennia any chance for the species of leaving the last suitable microrefugia, resulting in the two current rear edge populations.

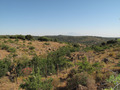

## 1 INTRODUCTION

Most of the circumboreal Cenozoic relict plants had a past wide distribution and now are extinct in most of their former geographic range (Huang et al., 2015; Milne & Abbott, 2002). Being adapted to a warm and moist climate (Huang et al., 2015; Kozlowski & Gratzfeld, 2013), their original distribution area began to contract progressively due to the counteracting decrease of temperatures and the increasing frequency and intensity of aridity during the late Miocene and the early Quaternary (Hampe & Jump, 2011; Suc et al., 2018; Svenning, 2003; Thompson, 2020). Many elements of this flora (e.g., *Davidia*, *Hemiptelea*, *Metasequoia*, *Gleditsia*, *Hamamelis*, *Juglans*, *Forsythia*, *Liquidambar*, *Parrotia*, *Pterocarya,* and *Zelkova*) survived as relicts in disjunct and/or isolated refugial areas in southeastern and western North America, East Asia, and Southwest Eurasia which experienced relatively long‐term stable climatic conditions up to present time (Huang et al., 2015; Kozlowski & Gratzfeld, 2013; Milne & Abbott, 2002; Tang et al., 2017; Zhang et al., 2017). In particular, in the European continent, Cenozoic mesophilous and thermophilous taxa persisted in the Iberian Peninsula, in Transcaucasia and on the largest Mediterranean islands (Médail, 2017; Thompson, 2020). Together with the two other main south European (Italian and Balkan) peninsulas, these areas played a major role for the survival of relict species, especially during the Pleistocene glacial–interglacial cycles (Dobrowski, 2010; Médail & Diadema, 2009; Quézel & Médail, 2003; Svenning et al., 2008).

Because climate has been undoubtedly the major factor responsible for range retraction and extinction processes, the members of the so‐called Palaeogenic flora have also frequently been named “climate relicts.” Climate relicts have been defined by Hampe and Jump (2011) on a population basis and are defined as “those populations of a species that persist in isolated enclaves of suitable climate space surrounded by areas with climatic conditions that do not permit the existence of the species”. Indeed, as climate relicts faced long‐term processes related to the complex dynamics of distribution patterns and survival chances (e.g., habitat fragmentation, isolation, local extinction, etc.), they often represent an optimal target to investigate the effect of ongoing climate change at population and species levels across large spatial and temporal scales (Woolbright et al., 2014).

The concepts of climate relicts and refugia are strictly related one to another. Conventional refugia have been most often referred to large‐scale glacial refugia (or macrorefugia) occurring at low latitudes for temperate species during the Pleistocene glacial periods. However, in recent years this view has significantly widened (Nieto Feliner, 2011), since the additional concept of northern interglacial macrorefugia has been largely recognized as well, along with the implication of microrefugia (and/or cryptic refugia sensu Stewart et al., 2010) for species persistence and post‐glacial colonization in face of shifting climate (Ashcroft, 2010; Mee & Moore, 2013; Rull, 2009, 2010; Stewart et al., 2010). In particular, microrefugia played a crucial role in supporting small isolated low‐density populations of species thriving beyond their reconstructed range boundaries, thanks to substantial decoupling of site climate from regional climate (Dobrowski, 2010; Keppel et al., 2012; Rull, 2009). With regards to that, topographic niche heterogeneity issuing from varied patchy habitats and steep microclimatic gradients represents a key factor for relict survival (Hampe & Jump, 2011; Hampe & Petit, 2005; McLaughlin et al., 2017). Accordingly, focusing on the distinctive characters of some populations (i.e., size, clustering type and geographical patterns), microrefugia were efficiently outlined by Rull (2009) as “small areas with local favorable environmental features, in which small populations can survive outside their main distribution area (the macrorefugium), protected from the unfavorable regional environmental conditions.”

Up to now, the spatial patterns and functioning mechanisms of microrefugia in buffering large‐scale climate are still rather poorly understood (Dobrowski, 2010; Rull, 2009). For instance, in the current interglacial period, water supply may have been the most limiting factor for mesophilous relicts in arid regions; hence, small scale convergent environments (e.g., valley bottoms, local depressions, streams, sinks) may have acted as suitable microrefugia (Dobrowski, 2010; McLaughlin et al., 2017). However, soil moisture availability may differ significantly even across a topographic gradient of few meters or at varying depths underground (e.g., Ashcroft & Gollan, 2013; Hylander et al., 2015; Le Roux et al., 2013), biasing interpretations at a fine‐scale level. Therefore, evaluations about the precise role of soil moisture within microrefugia deserve more accurate investigations (Ashcroft et al., 2012; Hampe et al., 2013).

Due to its complex biogeographical patterns and intriguing evolutionary history (Kozlowski & Gratzfeld, 2013; Naciri et al., 2019), the genus *Zelkova* is one of the most prominent among the Cenozoic climate relicts. It is commonly accepted that its origin dates back to approximately 55 Ma in the northern Pacific area (Burnham, 1986), although recent papers indicate northeastern China as the center of origin of *Zelkova* (Zhang et al., 2017). Wherever it originated, the members of this genus must have evolved under more or less constantly warm and humid climatic conditions. At present, the remnant species belonging to the genus *Zelkova* are distributed in two disjunct refugial areas with different climate types: Southeastern Asia, with *Z. serrata* (Thun.) Makino, *Z. sinica* C. K. Schneid. and *Z. schneideriana* Hand.‐Mazz. (China, Korea and Japan), and South‐Western Eurasia, harboring *Z. carpinifolia* (Pall.) K. Koch (Transcaucasia and Middle East), *Z. abelicea* (Lam.) Boiss. (Crete Island, Greece) and *Z. sicula* Di Pasq., Garfì & Quézel (Sicily Island, Italy) (Kozlowski & Gratzfeld, 2013). While the Southeastern Eurasiatic species still occupy part of the pristine distribution range of the genus, the current distribution of the westernmost taxa has been undoubtedly shaped by the climatic fluctuations of the past, with special regards to the Quaternary glacial–interglacial cycles (Kozlowski & Gratzfeld, 2013; Suc et al., 2018). Moreover, the two Mediterranean island species, *Z. abelicea* and *Z. sicula*, currently thriving on the montane‐ and thermo‐Mediterranean bioclimatic belts respectively, show that the ecological amplitude of many so‐called “temperate” relict species may have been underestimated (Médail, 2017). With respect to that, Svenning (2003) assumed, indeed, that relictual genera, such as *Pterocarya* and *Zelkova*, are cold‐sensitive as the extinct ones but relatively drought tolerant.

In Sicily, where the genus *Zelkova* was rather widespread until ca. 20,000 BP just before the Last Glacial Maximum (Sadori et al., 2008), nowadays *Z. sicula* occurs with only two tiny and critically endangered populations (Kozlowski et al., 2018). The extremely peculiar characteristics of both microrefugia hosting these relict nuclei, coinciding with the catchments of two small seasonal streams (cf. Figure 2a), have been noticed and highlighted since their discovery (Di Pasquale et al., 1992; Garfì et al., 2011). However, up to now no detailed data were available about the environmental fine‐scale processes favoring the survival of both populations. To improve the knowledge about such processes, topographical indexes, that is, the Topographic Position Index and the Topographic Wetness Index (Copeland & Harrison, 2015; Le Roux et al., 2013), were combined with the diachronic analysis of current soil moisture dynamics (Soylu et al., 2014) to better understand the role of microtopography in determining the existence of present‐day microrefugia. Plant growth and spatial patterns were additionally considered in order to (a) detect any fine‐scale differences between the two microrefugia locations which could play a role in sustaining the respective relict populations and (b) provide further clues on the possible ecological amplitude of the target species and inherent processes explaining its persistence.

## 2 MATERIALS AND METHODS

### 2.1 Study area

The two populations of *Z. sicula* (hereinafter named ZS1 and ZS2) are located on the northern slopes of Iblei Mts. (southeastern Sicily), ca. 17 km distant from one another between 315 and 530 m a.s.l. (Garfì et al., 2011). The regional climate is typically Mediterranean, with pronounced summer drought; annual rainfall is about 800 mm, and mean yearly temperature is 16.3°C (weather station of Melilli, 310 m a.s.l.). The sites hosting the two populations lie at the boundary between the thermo‐ and meso‐Mediterranean bioclimatic belts (Garfì et al., 2011).

The geomorphology of the entire Hyblaean area consists of an alternation of highlands forming a calcareous plateau and canyons, sometimes characterized by very steep slopes. Basalts issuing from submarine Plio‐Pleistocene eruptions represent the outcropping lithotypes in both the investigated sites (Carbone et al., 1987), and the prevailing soils are andic brown soils and lithosols where rock outcrop is abundant (Fierotti, 1988).

The landscape is characterized by a patchy mosaic of open woodlands and pasturelands, alternating with food and forage crop fields (Garfì et al., 2011). The past forest vegetation is testified by the frequent occurrence of scattered nuclei of cork oaks *Quercus suber* L., wild olives *Olea europaea* L. var. *sylvestris* (Mill.) Lehr. and Virgilian downy oak *Quercus virgiliana* Ten., whereas *Pyrus spinosa* Forssk., *Sarcopoterium spinosum* (L.) Spach, *Cytisus infestus* C. Presl and *Phillyrea latifolia* L. abound in the shrub layer. The present vegetation patterns issue from centuries of anthropogenic impact, involving coppicing and, above all, wildfires and grazing. Moreover, large areas have been cultivated in the past and their abandonment in the last decades triggered secondary succession processes, whose speed is strongly hampered by frequent disturbance due to cattle overgrazing and recurrent wildfires.

In order to compare on a quantitative basis the landscape matrix surrounding the two microrefugial areas, an analysis of local vegetation units was carried out through QGIS v.3.12 software, using data layers from the habitat maps (modified) available within the Management Plans of Natura 2000 sites of Sicily Region (http://www.artasicilia.eu/old_site/web/natura2000/index.html), inspired by the Corine Biotopes classification. The analysis was performed on the landscape unit of one square kilometer (1 × 1 km) surrounding each of the two microrefugia.

### 2.2 Field data collection and analysis

The complete inventory of the two populations was carried out by recording the position of each living plant through high‐resolution GPS device (Trimble XT 2005 Series, post‐processing accuracy 30 cm). To facilitate future monitoring, each inventoried tree was also numbered and labeled. Since exclusive clonal propagation was already assumed for the species (Garfì et al., 2011), we considered as inventory unit the tallest stem in a clump that was at least 50 cm afar from its nearest neighbor. Due to the remarkable heterogeneity of local spatial and topographical patterns (Garfì et al., 2011), population ZS2 was split in two subpopulations — ZS2a and ZS2b — that were treated separately in further data analysis and discussion.

In order to analyze plant growth performances in each of the three population units, the height of a subset of individuals was recorded. Sampling criteria were different, according to tree spatial patterns and population size. In populations ZS1 and subpopulation ZS2b, which show a scattered plant distribution, the measured individuals were selected through a systematic approach, that is, taking into account one out of four trees included in the population inventory; respectively, 66 and 31 individuals were considered. Within subpopulation ZS2a — which is much larger and thicker than the two others and shows a uniform linear distribution pattern — two sample plots (20 × 15 m and 20 × 10 m, respectively) were identified, one at the upstream and one at the central part of the surface occupied by the whole subpopulation; subsequently, the total number of the plants included in both the selected sample plots was measured (132 and 107 trees, respectively).

Mean height was calculated, and to highlight differences in growth potential among the three population units, ANOVA was carried out (R Core Team, 2020) including only trees taller than 100 cm. In addition, the top height, here defined as the average of the five tallest trees in each population unit, was used as quality index to assess the site productivity (Fu et al., 2018; Vanclay, 1992; Vanclay & Henry, 1988) under the different microrefugia conditions. Student's *t* test for two samples was performed in R (Mangiafico, 2015) to point out pairwise statistically significant differences.

Based on position data from the inventory, the Nearest Neighbor (NN) analysis was carried out through QGIS v.3.12 software. NN analysis is an effective tool to characterize tree stand structure; it is based on the index of aggregation CE of Clark and Evans (1954), which is a measure of the extent to which the observed distribution of the individuals of a given population differs from the theoretical random pattern. The value of CE may range between 0 (extremely clustered distribution) and 2.1491 (perfect equilateral triangle pattern) (Neumann & Starlinger, 2001; Pommerening, 2002). Values of CE = 1 are typical of random distribution, while CE > 1 and CE < 1 respectively indicate a trend to regularity or clustering. A test of significance for the deviation from random distribution was estimated using the *Z* test (Pommerening, 2002).

### 2.3 Topographical attributes

The tree position data were used to determine the extent of the microrefugial area concerning each of the three population units. These data represented the base for the subsequent topographical analyses. Calculations were carried out through QGIS v.3.12 by drawing a buffer area around each single tree, whose radius was arbitrarily fixed to 5 m after considering the plant distribution patterns and their position with respect to the microrefugia; dissolving of the output geometries provided the final microrefugia polygons. In order to distinguish between “plant population” and the respective “population microrefugium” hereinafter the initials “Pp” and “Pm” will refer to the three population unit acronyms ZS1, ZS2a and ZS2b.

Fine‐scale topographical analyses were carried out through QGIS v.3.12 employing a Digital Elevation Model (DEM) with a 2 m spatial resolution. The following topographical attributes were computed: elevation, aspect, slope, Topographic Position Index (TPI), and Topographic Wetness Index (TWI). All five descriptors were calculated at the microrefugium level for each of the three population units PmZS1, PmZS2a, and PmZS2b; in addition, TPI analysis was also carried out to compare the landscape matrix on the one square kilometer (1 × 1 km) area surrounding the microrefugial areas.

TPI is a very powerful metric to classify landforms (Weiss, 2001; see also Jenness, 2006, for technical details), providing useful diagnostic elements for a broad array of research fields, for example, geomorphology, hydrology, agronomic sciences, behavioral ecology, forest management, etc. (Copeland & Harrison, 2015; De Reu et al., 2013; Skentos, 2017; Tagil & Jenness, 2008). Due to its effectiveness in subdividing the landscape into morphological categories depending on topography, it can help identify — for instance — the topographic preference or habitat suitability for plants species (Han et al., 2011). TPI is calculated from the difference between a cell elevation value and the average elevation of the neighborhood around that cell. Positive values indicate that the cell is higher than its surroundings, while negative values means that it is lower. Topographic position is a naturally scale‐dependent feature. At a large scale, the topographic position of one point would be a ridge, whereas at a fine scale, the same point could be the bottom of upland drainage, which may be more meaningful for overall hydrological processes and/or micro‐climate (Jenness, 2006). When combining TPI at a small and large scale and including slope data, 10 landform classes can be distinguished, from “Deeply incised streams” to “Mountain tops” (cf. Figure 2 for the complete list) (Weiss, 2001). In our analysis, in the aim of bringing out even the smallest hollow trails but also avoiding misrepresentation of the major landforms, we considered appropriate a fine‐scale radius of 10 m and a large‐scale radius of 250 m.

TWI is a measure of available soil moisture of a given site in the landscape and is calculated as ln(*α*/tan*β*), where *α* is the specific upslope catchment area and *β* is the local surface slope, assuming steeper slopes offer less opportunity for water retention (Le Roux et al., 2013; Mattivi et al., 2019). It predicts groundwater occurrence (as channels, seeps, or riparian water tables) in convergent topographies with large upslope catchment areas and at the foot of hillslopes where slope gradients drop dramatically (McLaughlin et al., 2017). Larger upslope drainage areas and shallower slopes will produce larger TWI values which indicate higher soil moisture content. TWI is frequently used as a proxy of soil moisture at fine spatial scale in ecological studies (Kopecký & Čížková, 2010; Le Roux et al., 2013). In our study, we applied it to highlight differences among the microrefugial situations. In order to facilitate the comparison, the output real TWI values were reclassified in ten integer unit classes, ranging from 2 to 11.

### 2.4 Soil moisture measurements

To evaluate the possible influence of groundwater on the maintenance of the investigated relict populations, real soil moisture accessibility by plants and its variation through time were analyzed (McLaughlin et al., 2017; Miller et al., 2010). Measurements of volumetric water content (VWC) were carried out in each of the two refugial sites by weather stations (WatchDog 2000 Series Weather Station, Spectrum Technologies Inc.) installed in the proximity of the main streams. Data collection included soil moisture at three different depths (0, 30, and 60 cm, respectively) with VWC probes buried as close as possible to the stream bottom; additionally, precipitation, air temperature, and air humidity were recorded, too. Recording was done at 30 min temporal resolution and lasted from April 8, 2014 to December 31, 2017 on ZS2, and March 8, 2018, on ZS1. Data other than VWC were not used in the present research, but supported the correct interpretation of local climatic records and eventually confirmed the opportunity to discard some aberrant values due to episodic malfunctioning of soil probes.

Analysis of VWC focused on key months with respect to either regional climate patterns or the phenology of the target species. Therefore, we selected the mean monthly data of January (the rainiest month), April (the start of plant growing and the last month prior the dry season), July (the driest month), and September (the resumption of autumn rains and of a possible additional growth flux).

## 3 RESULTS

### 3.1 The landscape matrix

**FIGURE 1:**
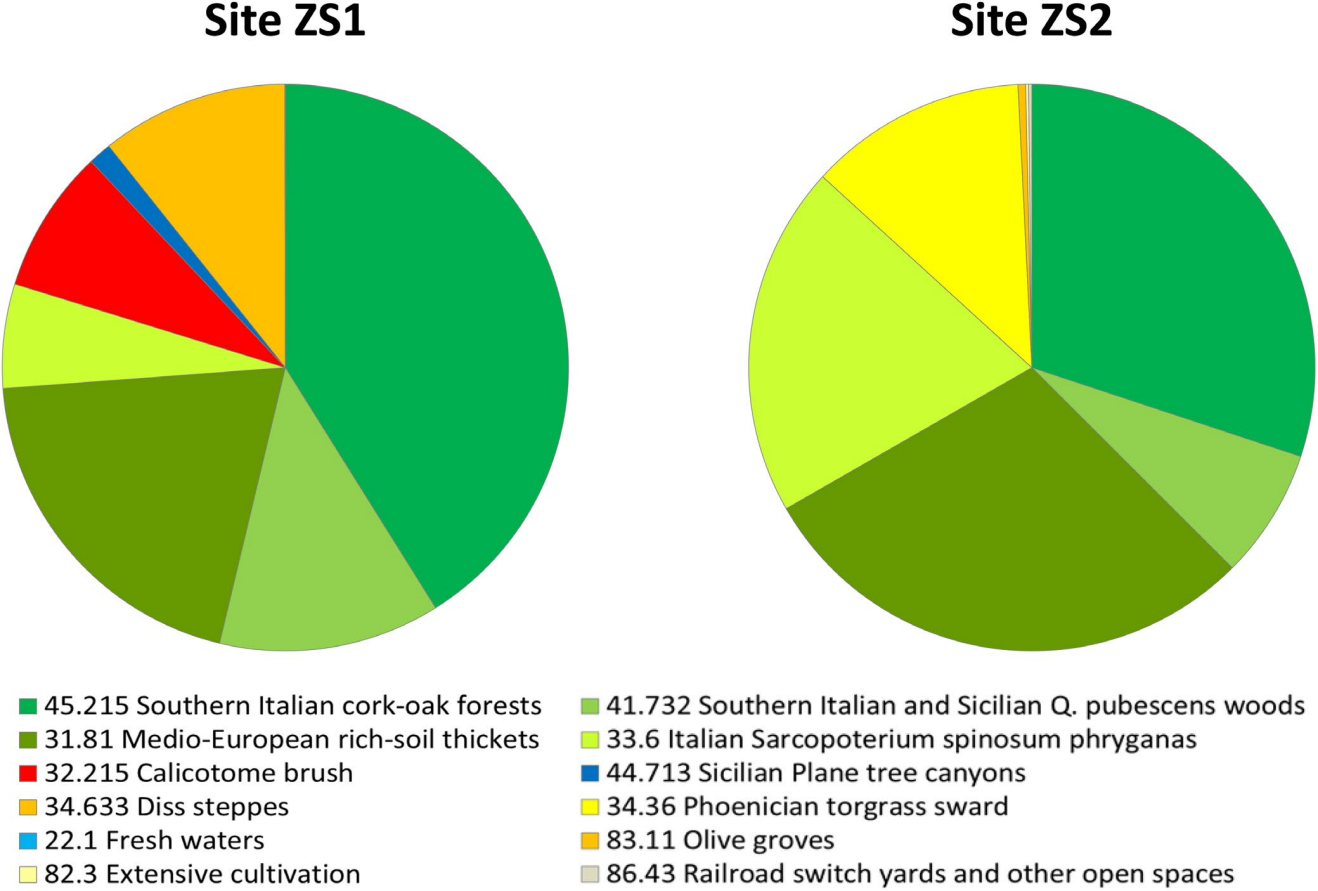
Habitat area partitioning in the two investigated landscape units. In each site data refers to a 1 square km surface (1 × 1 km)

At the landscape scale, the overall vegetation cover patterns of ZS1 and ZS2 areas are quite similar and can be referred to thermo‐xerophilous evergreen sclerophyllous maquis communities of the Sicilian thermo‐(meso‐)Mediterranean belt (Garfì et al., 2011). The cumulated surface of four shared habitats (namely 45.215*‐Southern Italian cork oak forests*, 41.732*‐Southern Italian and Sicilian* Q. pubescens *woods*, 31.81*‐Medio‐European rich‐soil thickets* and 33.6*‐Italian* Sarcopoterium spinosum *phryganas*; see also Appendix 1) accounts for 79.8 and 86.8% of the entire extent of ZS1 and ZS2 sites, respectively (Figure 1). Scattered cork oak stands are the dominant forest type in both locations, covering more than 41% on ZS1 and 30% on ZS2. The only distinctive difference concerns the relative abundance of perennial grasslands, represented in ZS1 by habitat *34.633‐Diss steppes* (10.7%), and in ZS2 by habitat *34.36‐Phoenician torgrass sward* (12.5%).

**FIGURE 2:**
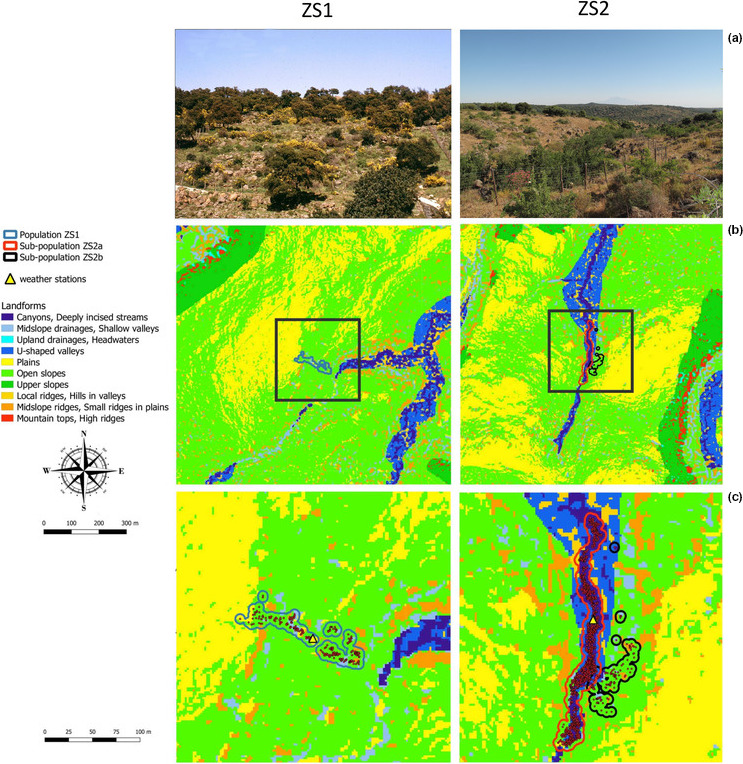
General view of the investigated sites (a) and maps of landforms in the two locations at the landscape scale (b) (grid 1 × 1 km) and the microrefugia level (c). In (a), PpZS2a is concentrated in the valley bottom, whereas PpZS2b consists of scattered plants on the right‐bottom side of the picture

**FIGURE 3:**
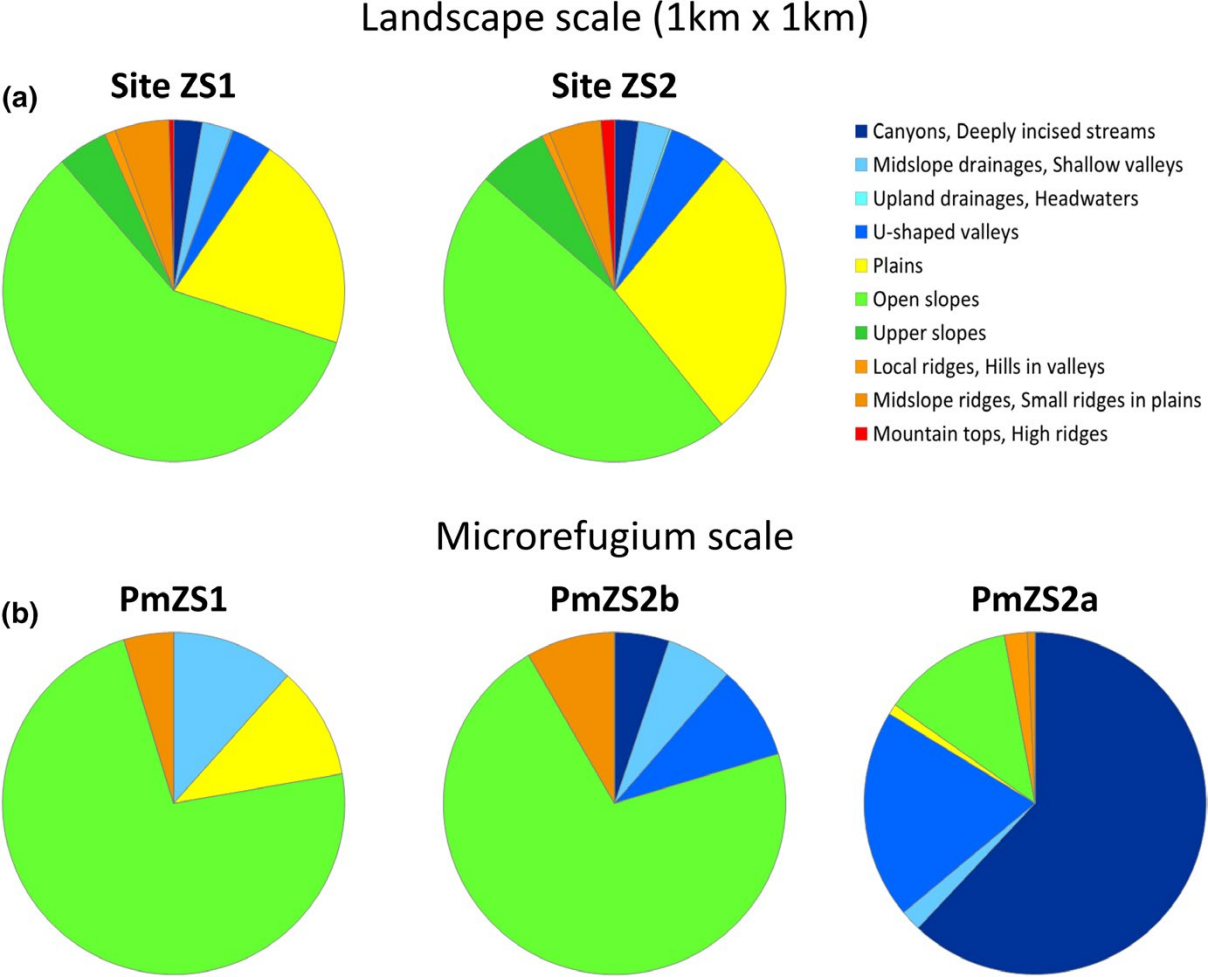
Landforms area partitioning at the landscape scale (a, grid 1 × 1 km) and the microrefugia level (b)

The same pattern results from the TPI analysis. Landforms in the two locations are very similar (Figure 2b), with a large predominance of “Open slopes” and “Plains”, respectively representing 58.7% and 20.4% on ZS1, and 47.1% and 28.3% on ZS2 (Figure 3a). Convergent topographies (i.e., “Canyons/Deeply incised streams”, “Midslope drainages/Shallow valleys”, “Upland drainages/Headwaters” and “U‐shaped valleys”) totalize about 10% of the analyzed area in both sites (Figure 3a); among them, “U‐shaped valleys” attain the highest rate, with 3.8% in ZS1 and 5.5% in ZS2.

### 3.2 Topographical attributes at microrefugium level

The three current population units PpZS1, PpZS2a, and PpZS2b are extremely small. The 5‐m buffering of plant position provided the surface of the three corresponding microrefugia that measured only 2,896, 4,852, and 2,244 m^2^, respectively.

Differently with respect to landscape matrix level, landform patterns are remarkably distinct; those differences are particularly evident between PmZS2a and the two other microrefugial locations (Figure 2c). In the former, convergent topographies, namely “Canyons/Deeply incised streams,” “Midslope drainages/Shallow valleys,” and “U‐shaped valleys,” are largely predominant, overall totalizing almost 84% of the whole microrefugium area (Figure 3b); only ca. 20% of the same categories characterizes also PmZS2b, but mainly concentrating on a small shared area with PmZS2a (Figure 2c). Conversely, in PmZS1 the sole TPI class referable to convergent environments is “Midslope drainages/Shallow valleys” and occurs on only 11.6% of its surface (Figure 3b). Furthermore, as a whole PmZS1 and PmZS2b appear to be somehow similar due to the large prevalence in both of them of “Open slopes,” occupying about 73% and 71% of the respective area (Figure 3b).

**FIGURE 4:**
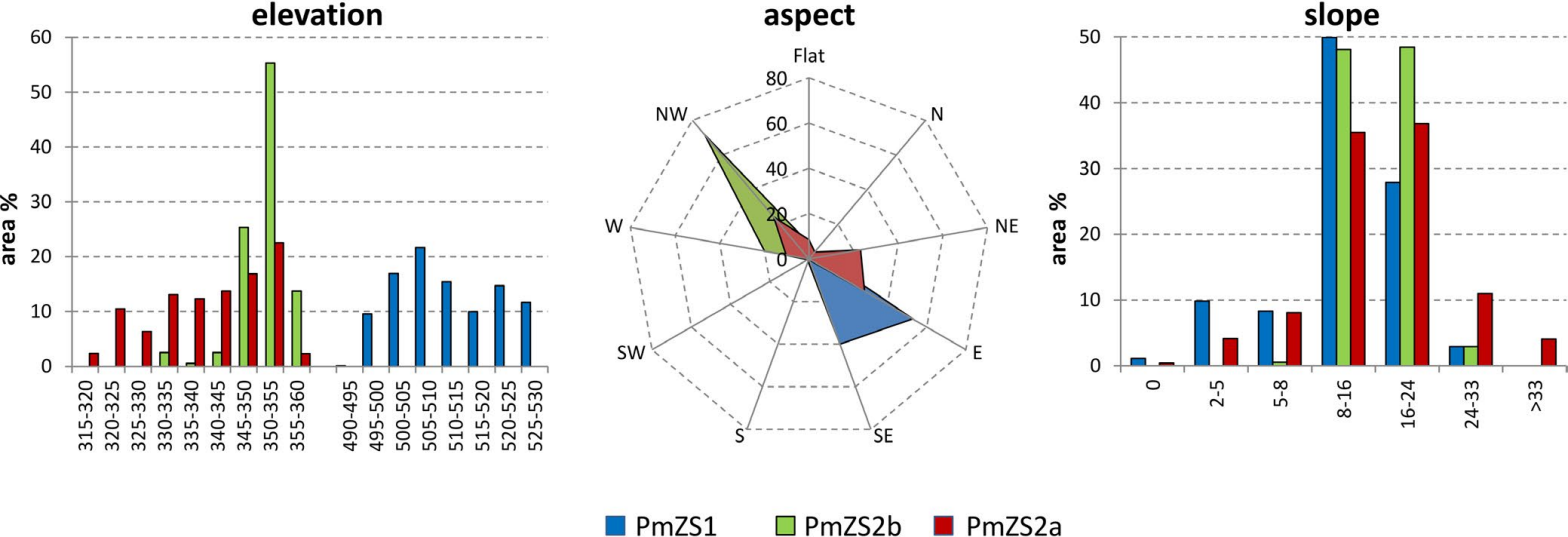
Area partitioning in % of the basic topographical attributes in the three microrefugial locations. Elevation is in m a.s.l. Slope is in degrees

Some differences among the microrefugial settings can be highlighted also with concern to the basic topographical attributes (Figure 4). Overall, PmZS2 altitude ranges from 315 and 360 m a.s.l., with around 80% of total area in PmZS2b and 40% in PmZS2a lying between 345 and 355 m a.s.l.. By contrast, altitudinal values are rather higher in PmZS1, with the greatest surface (38.6%) comprised from 500 to 510 m a.s.l., and extreme elevations spanning from 490 to 530 m a.s.l.

The surfaces related to PmZS1 and PmZS2b are nearly opposite in terms of aspect: the former is almost completely E‐SE oriented (ca. 93% of total area), whereas the latter has a prevailing orientation (about 92%) at NW‐W and only 6.8% of flat surface. PmZS2a, which occurs largely in the bottom of the ravine, is instead characterized by a more variable aspect, with more than half (51.4%) facing E‐NE, 34.6% NW‐W and 8.5% lying on flat position.

Slope is commonly steep in all microrefugial locations. From 35.5% (PmZS2a) to 49.9% (PmZS1) of land can be classed as “hilly” (slope 8 to 16 degrees), whereas 27.9% (PmZS1) to 48.5% (PmZS2b) is “moderately steep” (slope 16–24 degrees). A significant rate (ca. 15%) of steepest slopes can be observed particularly in PmZS2a.

**FIGURE 5:**
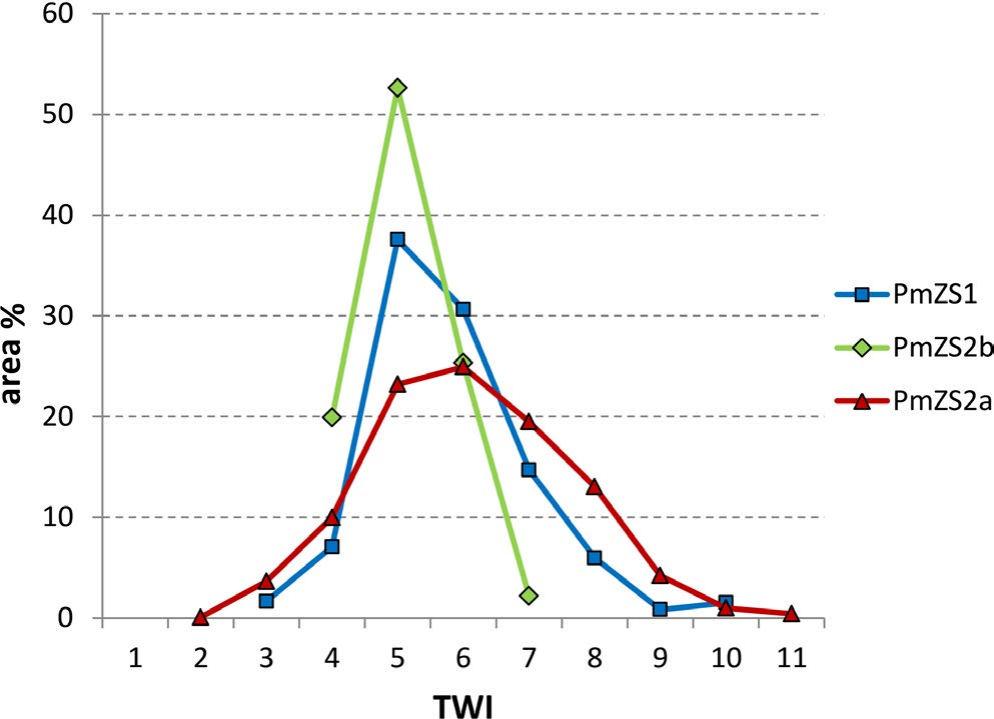
Area partitioning of the three microrefugia in Topographic Wetness Index (TWI) classes

As far as the potential soil water content, in the three investigated microrefugia most of surface falls within the two TWI intermediate classes 5 and 6, but with varying cumulative rate (Figure 5): 68.2% in PmZS1, 48.1% in PmZS2a and 77.9% in PmZS2b. Higher class values, from 7 onward, are poorly represented in PmZS2b (2.2%), whereas they are relatively frequent in PmZS1 (23.0%) and, even more, in PmZS2a (38.2%).

### 3.3 Seasonal dynamics of soil moisture

**FIGURE 6:**
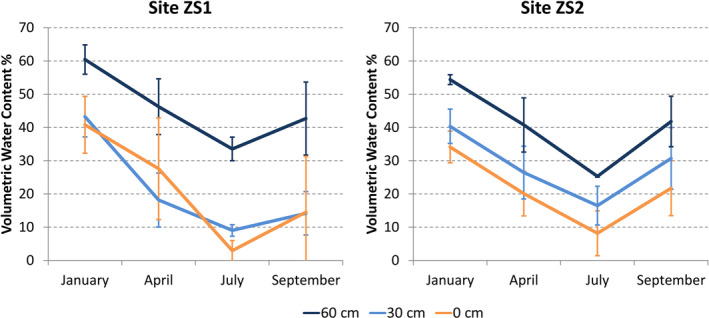
Volumetric Water Content (VWC) variation through the year in the two investigated sites. Bars indicate ± *SD*

The general trend of volumetric water content is similar in both sites, with top VWC in January, a progressive decline in April and July, and a following increase in September (Figure 6). However, a prominent fact can be observed in summer. Despite July corresponds to the peak of summer drought when precipitations are very low (or even null), in this month soil moisture at 60 cm depth never decreases below 25% in site ZS2, with values exceeding 33% in site ZS1. Interestingly, at lesser depths this pattern is inversed, with VWC of July higher in ZS2 than in ZS1 at both 0 and 30 cm; the same pattern was observed also in September and April, but in the latter only at 30 cm depth. Nevertheless, in both sites VWC in July at lower depth attains values exceeding the soil moisture content in April at upper depths.

### 3.4 Stand growth and spatial patterns

**FIGURE 7:**
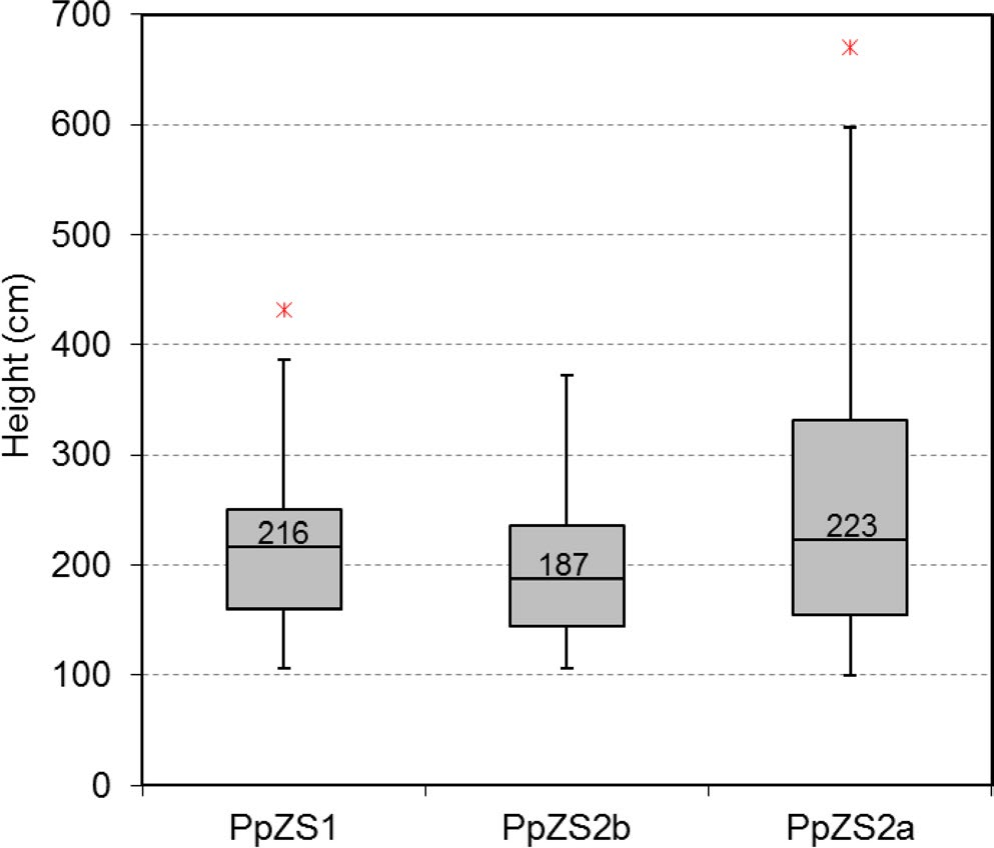
Boxplot of height variation among the three investigated populations PpZS1, PpZS2a, and PpZS2b. Box size represents the interquartile range, the black line is the median, the whiskers indicate variability outside the upper and lower quartiles, and individual points are outliers (only the min and the max values are displayed, if any)

Plant growth attributes allowed inferences about the site quality index. Plant height achieves the greatest values in PpZS2a and the lowest in PpZS2b, with an average above 2.50 m in the former and not exceeding 2.00 in the latter (Table 1). The boxplot outlines a similar setting, but it points out remarkable differences in the range width of boxes. In particular, the upper quartile in PpZS2a is taller than 3.30 m, whereas in the other stands, it barely achieves 2.50 m (Figure 7). Moreover, only in PpZS2a the upper whisker largely surpasses 4.00 m (5.97 m), with outliers up to 6.70 m. ANOVA showed that differences in plant height among the three population units are significant at *p* < 0.05. The top height index confirms this trend, with values in PpZS2a about over 2.00 m greater than in the better of the two other populations (Table 1). Student's *t* test showed that also top height differences between PpZS2a and the two other population units are significant (*p* < 0.01), while non‐significant difference is found between PpZS1 and PpZS2b.

**TABLE 1:** Biometric and spatial indices related to the investigated populations

Pop	No. trees total	Biometric indices				Spatial indices			
		Sampled trees (retained)	Mean H (*SD*)	Median *H*	Top *H* (*SD*)	Observed distance	Expected distance	NN index	*Z*‐score
PpZS1	262	66 (56)	215.2 (69.0)	216	362.8^a^ (53.2)	1.03	2.88	0.36^a^	−19.80
PpZS2a	1,426	239 (181)	250.8 (120.8)	223	575.8^b^ (62.2)	0.59	1.40	0.42^a^	−41.68
PpZS2b	122	31 (20)	196.7 (68.4)	187	294.8^a^ (42.9)	2.09	4.10	0.51^a^	−10.36

Note

Significant differences (*p* < 0.01) of top height (top H) are indicated with different letters; Z‐score is significant at *p* < 0.01 at all population units.

NN index ranges from 0.36 in PpZS1 to 0.51 in PpZS2b and is all significant at *p* < 0.01 (Table 1). Though CE values are a bit different from one another, all of them indicate a more or less pronounced clustered distribution.

## 4 DISCUSSION

### 4.1 Not only a troubled history, but also effective adaptations to cope with a deteriorating climate

**FIGURE 8:**
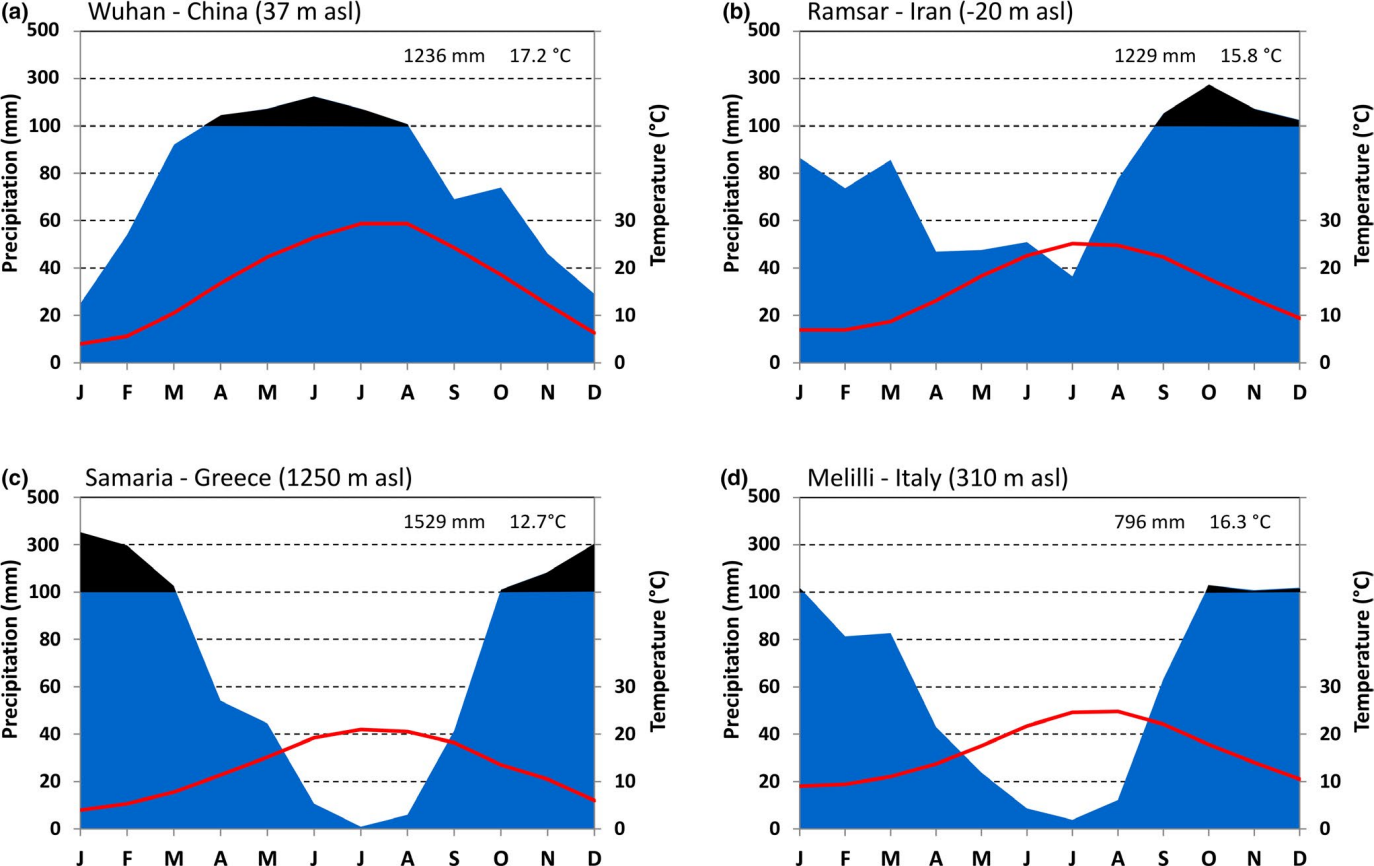
Climatic diagrams referred to localities representative of the range of the easternmost *Zelkova* species (a), the Transcaucasian *Z. carpinifolia* (b), the Cretan *Z. abelicea* (c), and the Sicilian *Z. sicula* (d)

Relict plants may provide very useful indications to elucidate aspects related to species extinctions, relying on potential characters that enabled some taxa to survive regional or lineage disappearance (Gavin et al., 2014; Grandcolas et al., 2014). According to this statement and also due to its outstanding biogeographic diversification (Kozlowski & Gratzfeld, 2013; Zhang et al., 2017) and complex evolutionary history in face of past climate fluctuations (Magri et al., 2017; Naciri et al., 2019), the genus *Zelkova* represents a very suitable model to investigate the ecological amplitude within a relict taxonomic group, as well as the complex nature of marginality in plants (Abeli et al., 2014). The remnant representatives of this genus currently occur in quite distinct climatic regions. The three easternmost species thrive under temperate and humid climates with no summer drought (Figure 8a), very similar to the Cenozoic subtropical environments where the genus originated and spread throughout Eurasia (Kan‐Kan et al., 2016; Zhang et al., 2017). As for the species forming the western disjunct range, *Z. carpinifolia* grows under almost constantly moist climatic conditions, with a reduction of summer precipitations but a very short dry season (Figure 8b). Differently, both Mediterranean species *Z. abelicea* and *Z. sicula* live under typical Mediterranean climates, with 3–4 months of summer drought. However, due to their different altitudinal location, these two species are subject to sharply different rainfall regimes, so that the Sicilian populations enjoy only half of the total yearly rainfall and thrives in areas subject to 3.6°C higher mean annual temperature with respect to the Cretan ones (Figure 8c,d).

Huang et al. (2015) pointed out that more than temperature, the annual precipitation regime and the short endurance of the dry season are the factors which appear more strictly correlated with the modern distribution of Cenozoic relict taxa. As a consequence, in southeastern Asian regions, the relative stability through time of yearly humid conditions, in addition to complex topography, greater habitat variety, and a limited impact of Quaternary glaciations may explain the persistence of such a rich and diverse relict flora to which *Zelkova* belongs (Huang et al., 2015; Zhang et al., 2017). Conversely, in Southwest Eurasia, the current distribution of *Zelkova* species was strongly shaped by the shift from a moist warm–temperate climate to summer–dry patterns during the Middle Pliocene (ca. 3.2 Ma), which turned into even greater dryness during the glacial periods, from 2.3 Ma onwards (Suc et al., 2018). Such a contrasting climate, along with the presence of east‐west oriented physiographic barriers (e.g., the Pyrenees, the Alps and the Mediterranean Sea) and restricted dispersal effectiveness (Certini et al., 2020; Svenning & Skov, 2007) remarkably limited the migration and recolonization chances for *Zelkova* species and were responsible for their step‐wise massive extinction at higher latitudes (Magri et al., 2017; Suc et al., 2018). Rather common in Central Europe at the Mio‐Pliocene boundary up to Late Pliocene (e.g., Kvaček et al., 2019; Teodoridis & Kvaček, 2015), *Zelkova* is still attested during the Middle Pleistocene as relatively frequent at lower latitudes in the northwestern Mediterranean, except in the westernmost Iberian peninsula where it was already absent in the Early Pleistocene (Postigo‐Mijarra et al., 2010). The harsh cold and dry conditions of glacial times involved its progressive rarefaction in Southern Europe, and from the Middle Pleistocene onwards only Italy and Greece continued to host large forest communities with *Zelkova* (or *Ulmus/Zelkova*), especially during the interglacial stages (Magri et al., 2017). Within the whole continental Europe, the Eemian interglacial (130–115 ka) was the last stage when *Zelkova* was still present in the Balkan area though in very small and sparse amounts (Sinopoli et al., 2018), and rather common in the Italian peninsula (Bertini, 2010), becoming definitively extinct around 30 ka in Central Italy (Follieri et al., 1986).

In the global view, it seems obvious that the latitudinal factor played a major role in shaping the occurrence of large‐scale refugia for *Zelkova* in glacial periods. Nevertheless, it is undeniable that landscape niche heterogeneity largely concurred to preserve these mesophilous forest tree species especially during interglacials (Médail & Diadema, 2009; Dobrowski, 2010), thanks to the occurrence of locally more favorable conditions (e.g., owed to altitude, aspect, convergent topography, etc.) (Hampe & Jump, 2011; McLaughlin et al., 2017). On the other side, such variability confined relict taxa to very peculiar and spatially delimited locations. Accordingly, the two Mediterranean highly relictual trees *Z. abelicea* and *Z. sicula* remained “trapped” within their refugial niches on Crete and Sicily, respectively, at high elevation (Bosque et al., 2014; Fazan et al., 2017), or in topography‐driven mesic environments (Garfì et al., 2011; Goedecke & Bergmeier, 2018; Kozlowski et al., 2014) such as hydrological microrefugia, relatively decoupled from the regional climate.

A condition for a moist site to act as a hydrological refugium depends on both the spatial‐temporal variability of water accessibility and its matching with the ecological traits of the target species (McLaughlin et al., 2017). As a matter of fact, paleoecological studies point out that the majority of the taxa that went extinct during Quaternary glaciations were among the most thermophilous ones, whereas the relicts which survived within scattered refugia in Southern Europe were nearly as thermophilic as, but more able to tolerate drought stress than, the extinct ones. In fact, *Zelkova* and *Pterocarya* figure among the most drought‐tolerant representatives of the Cenozoic relict flora and were the last to disappear from continental Europe (Svenning, 2003). In addition to such inherent factors of the genus and to the peculiar safety role of microrefugia, it can be also speculated that the two modern Mediterranean *Zelkova* species could have either developed local evolutionary adaptations as a consequence of long isolation in microrefugia (whose environmental conditions are largely different than in macrorefugia) (Mee & Moore, 2013) or even derived from “ecological” speciation under strong adaptive selection in marginal habitats (Kiedrzyński et al., 2017; Nieto Feliner, 2011; Stewart et al., 2010). For instance, investigations on vein density in *Z. schneideriana* showed that this trait intensifies along with increasing precipitations, highlighting its important role in plant transpiration processes (Wang et al., 2016). Actually, in both Mediterranean *Zelkova* relicts these features, coupled to small leaf size, are stable characters (Denk & Grimm, 2005; Wang et al., 2001), indicating functional adaptations to withstand high levels of evapotranspiration during the growing season. Besides, in the Sicilian species, additional anatomical and morphological drought‐tolerance traits have been previously detected (Garfì et al., 2011) such as thick leaf mesophyll, granular wax ornamentation at the lower epidermis, densely haired leaf surface, abundance of mucilage in palisade tissue. Also phenotypic plasticity can be regarded as an adaptive feature to arid environments. The typically small size of *Z. sicula* trees in their current habitat has been considered as an adaptive response to thermo‐hydric stress involving size reduction (Abeli et al., 2014; Garfì et al., 2002), also in the light of evidences that a few plants cultivated in less constraining conditions exhibit the habit of veritable trees (Garfì & Buord, 2012; Garfì et al., 2011). Similarly, dwarfed individuals prevail in all populations of *Z. abelicea* and the frequency of this habit rises along a west–east precipitation decrease gradient, becoming exclusive in the easternmost scattered population of Thripti (Goedecke & Bergmeier, 2018; Kozlowski et al., 2014). The above‐mentioned manifold adaptive traits, associated with the effectiveness of hydrologic microrefugia in providing more “species‐specific” (McLaughlin et al., 2017) suitable conditions that buffer the stress linked to regional climate, can finally explain the persistence of *Z. sicula* in the current locations.

### 4.2 Hydrologic microrefugia: the last option?

As stated by Dobrowski (2010), to assess the climatic basis for microrefugia two factors must be carefully evaluated: (a) the traits of regional climate and their impact in limiting the species distribution, and (b) the appraisal of mechanisms that allow local topography to moderate regionally constraining climatic patterns, thus allowing for relicts to persist locally. At a wider scale, vegetation cover broadly mirrors the regional climate, and vice versa climate represents a major driving force determining local species assemblages. In the case of *Z. sicula*, the landscape matrix surrounding the investigated microrefugia is amazingly uniform in terms of landforms and, above all, in terms of composition and structure of vegetation patterns, with typically Mediterranean thermo‐xerophilous sclerophyllic communities predominating at both sites (Garfì et al., 2011). It is quite blatant how this picture appears so far away from the environmental settings within which the genus *Zelkova* originated. This issue provides outstanding clues of the paramount role of current microrefugia to allow the preservation of the highly relictual populations of *Z. sicula*.

Such extremely small microrefugial locations are wedged in, or anyhow typified by convergent topographies. It has been broadly recognized (e.g., Dobrowski , 2010; McLaughlin et al., 2017) that, in contrast to glacial stages, during interglacials like the current one, convergent environments may support microhabitats that are moister than the surrounding matrix, acting as effective microrefugia for mesophilous species. Studies on plant traits associated with topographic contrasts (Copeland & Harrison, 2015) have clearly pointed out that the trees strictly linked to cool–humid microclimatic conditions/topography are often located at the southernmost edge of the distribution range of the species or belong to genera with a northerly center of origin. The complex evolutionary history of *Zelkova* all along the past climatic fluctuations and the enclave restriction of *Z. sicula* at the low‐latitude limit of the genus range suggest that the Sicilian climate relict could be represented now by “rear edge” populations (Hampe & Petit, 2005). They supposedly have been left behind during the northward species range shift following the Last Glacial Maximum (Hampe & Jump, 2011), owed to low dispersal ability (Certini et al., 2020) in face of long‐distance recolonization or topographic/geographic barriers.

Contrarily to the landscape matrix, the three investigated hydrologic microrefugia show some structural differences. Compared to PmZS1 and PmZS2b, in PmZS2a convergent environments are much more diversified: deeply incised landforms (i.e., “Canyons/Deeply incised streams” and “U‐shaped valleys”) are largely prevailing herein, whereas shallower hollow trails (“Midslope drainages/Shallow valleys”) are almost exclusive in the other microrefugia (Figures 2b, 3b). The former landscape features are obviously more strictly related to moister microclimates (e.g., larger drainages versus more ephemeral streams) (Copeland & Harrison, 2015; Weiss, 2001), and potentially more suitable for supporting higher water plant uptake, buffering seasonal fluctuations in precipitation (Barbeta & Peñuelas, 2017; Miller et al., 2010). Also, more favorable conditions at PmZS2a in terms of moisture supply were confirmed by the presence of some hygrophilous elements referred to the classes *Nerio‐Tamaricetea*, *Molinio‐Arrhenatheretea,* and *Phragmito‐Magnocaricetea*, described in previous investigations (Garfì et al., 2011).

The assumptions above are consistent with results of topographical wetness analysis (TWI), which showed PmZS2a was on the whole theoretically more humid than the two others (Figure 5). Though with limitations (e.g., Kopecký & Čížková, 2010; Le Roux et al., 2013), TWI can provide good proxy data on soil moisture availability in ecological studies as it reflects fairly well the true humidity content, especially at the greater depth (Han et al., 2011). However, it can vary even in a ray of few meters; hence, for more reliable evaluations of qualitative variation through time quantitative fields measurements are recommended (Le Roux et al., 2013). Actually, multi‐annual recordings of VWC in both sites showed that soil moisture in July maintains relatively high and stable values at lower depth, overall even above the topmost soil moisture during spring season, when growth processes re‐start. According to that, it is guessed that the soil moisture regime can significantly attenuate summer drought stress, representing a critical factor enabling the survival of the target species on both an annual and perhaps long‐term basis (Barbeta & Peñuelas, 2017; Miller et al., 2010). Moreover, the basic role of groundwater in supporting terrestrial vegetation is extensively documented (e.g., Evaristo & McDonnell, 2017, for a review), and especially in xeric ecosystems plants exploit such favorable microenvironments worldwide, clearly demonstrating the importance of these sites to support locally unique species or plant assemblages in face of dry regional climates (McLaughlin et al., 2017).

Though not specifically evaluated in the present research, soil composition and texture may corroborate the effects of convergent topography, thus enhancing the function of hydrological microrefugia. As a matter of fact, the two investigated sites are quite identical from the geological (Carbone et al., 1987) and pedological (Fierotti, 1988) point of view, being dominated by andic brown soils issued from volcanic outcrops (basalts). Local soils are clayey (52% clay, 32% loam, 16% sand, personal observations from site PmZS1, unpublished) and present a high allophane content which improves their water holding capacity. Concerning soil texture or composition, we could not remark any striking difference between the valley bottom and adjacent hillslopes. Therefore, it is very likely that the average good capacity of water storage and retention of local soils can contribute to the water supply of the investigated relict populations during the arid season, but we guess that the effective functioning of microrefugia is mainly controlled by the micro‐topographical settings.

Interestingly, based on TPI and TWI patterns it was expected that VWC content in site ZS2 was always higher, whatever the depth and season. Conversely, this rule was only confirmed from April to September especially at 30 cm depth, whereas it was always greater at the lowermost layer in site ZS1 (Figure 6). The observed pattern may depend on the possible short distance moisture variability (Le Roux et al., 2013) in the investigated sites, as also suggested by different TWI values in the cells of recording probes, resulting in wetness class 8 at ZS1 and class 6 at ZS2 (data not shown). Whatever the reason, it is anyway very likely that in both sites, a conspicuous and crucial water supply is permanently available to plants at the lowermost soil layers even during the driest months.

As for the main topographic features, while the slope is relatively steep at all locations, and the differences in elevation are not particularly significant (ZS1 is on average about 150 m higher than ZS2), aspect differs among microrefugia. In fact, no clear preferential facing emerges; it is quite variable between PmZS1 and PmZS2, though the most extreme facings at either the cooler north or the warmer south are never represented (Figure 4). These settings, coupled to the topographic patterns discussed above, provide speculative issues about a relative ecological amplitude characterizing the Sicilian *Zelkova* relict. Similar findings, indeed, had already been reported for its closest Mediterranean relative, the Cretan *Z. abelicea* (Bosque et al., 2014; Goedecke & Bergmeier, 2018). Nevertheless, in spite of such plastic traits, extreme drought events can trigger a more or less severe decline, from leaves withering to shoot and even stem desiccation (Garfì et al., 2002), supporting the assumption that the current microrefugia probably lie at the extreme boundary of (if not beyond) the potential range of *Z. sicula*. According to that, the present refugial locations must be classed as “*relative”* hydrologic refugia (sensu McLaughlin et al., 2017), that is, microsites supporting refugia with a moisture availability different with respect to the surrounding landscape, but susceptible to experience critical shortage as a consequence of seasonality and regional climate instability. This issue can be of a major concern for conservation, especially within scenarios of ongoing global warming trends.

### 4.3 Site‐dependent population traits and implications of confinement in microrefugia

Species ecological amplitude and site productivity are inherently correlated variables: soil quality, climate patterns, topography features, and other factors may influence growth, mortality and recruitment (Vanclay, 1992), as well as the phenotypic plasticity of plant species (de Kroon et al., 2005). The main growth metrics of the investigated populations of *Z. sicula*, namely mean/median and top height, showed a significantly higher growth rate in PpZS2a than in the two other population units (Table 1, Figure 7). These results are consistent with information derived from analyses of topographic indices and confirm the better habitat suitability of PmZS2a, as already suggested by previous DCA investigations based on abiotic and biotic data matrices (Garfì et al., 2011). Phenotypic traits mirror quite well the main environmental refugial characteristics, and in the same time concur in highlighting the relative plasticity of the target species (de Kroon et al., 2005), apparently able to withstand also harsher environments (as in PmZS1 and PmZS2b) with relatively pronounced water deficit. However, the intrinsic growth potential of *Z. sicula* should have remained unknown unless some trees were grown for a relative long time‐span under climatic conditions which are by far more favorable than in the wild (cf. Garfì et al., 2011). Unexpectedly, an individual cultivated since 28 years at 820 ma.s.l. under cooler climate exhibited an annual growth rate of 40–50 cm, reaching the current height of about 11 m. Similar patterns are being observed also in specimens maintained ex situ in different botanical repositories (e.g., the Botanic Garden of Catania in Sicily, the Conservatoire Botanique National de Brest in France, the Regional Forest Nursery of Spinagallo in Sicily; G. Garfì, pers. obs.).

Spatial patterns attributes provide additional information on population dynamics and propagation processes. Nearest Neighbor indices reveal, at variable extent in the three population units, a pronounced clumped distribution of trees, which is typical of clonal populations especially proliferating by root suckers (Bittebière et al., 2020). Suckering is guessed as the exclusive asexual regenerative strategy in *Z. sicula* (Garfì et al., 2011) and can be stimulated or even amplified by external disturbance factors involving critical injury to plant body (Klimesǒvá & Martinkǒvá, 2004), such as stem decline observed after severe drought stress (Garfì et al., 2002, 2011). In the Sicilian relict, clonality had been already inferred by studies on genetic diversity carried out by nuclear and chloroplast DNA markers that displayed different molecular profiles between the two known populations but no intra‐population variation, suggesting a complete lack of sexual reproduction (Christe et al., 2014; Fineschi et al., 2004; Naciri et al., 2019). Additionally, clonal propagation and sexual recruitment suppression are quite consistent with the triploid condition (Eckert, 2002) reported for this species (Nakagawa et al., 1998), and most probably issuing from its putative hybrid origin (Christe et al., 2014; Denk & Grimm, 2005; Naciri et al., 2019). Clonality is a common feature for plant species living in marginal habitats and remnant plants (Abeli et al., 2014; Bittebière et al., 2020; Eckert, 2002; Honnay & Bossuyt, 2005; Kavecki, 2008). For instance, it has been widely documented for relict populations of *Olea europaea* L. subsp. *laperrinei* (Batt. & Trab.) Ciferri in the hyper‐arid Saharan mountains (Anthelme et al., 2008; Baali‐Cherif & Besnard, 2005), or for the single‐clone triploid population of *Lomatia tasmanica* W.M. Curtis restricted to the sides of few creeks in south‐western Tasmania (Lynch et al., 1998).

Clonal propagation represents most often a survival strategy for many plant species under suboptimal environmental conditions (Abeli et al., 2014), especially when the marginal nature of habitats is due to abiotic factors (Kavecki, 2008). One of the most important adaptive functional traits of clonality is clonal integration, involving very important ecological consequences in heterogeneous environments (Liu et al., 2016; Pennings & Callaway, 2000). Clonal integration consists in sharing resources between ramets of the same clone, in case some of them grow in harsh microsites with scarce resources and/or severe stresses/disturbances whereas others are located in more favorable microhabitats. In such cases, a translocation of resources from the less to the most disadvantaged ramets allows clonal plants to efficiently cope with environmental heterogeneity by alleviating local resource shortages, buffering environmental stresses and disturbances, supporting competitive ability and/or increasing invasiveness (Honnay & Bossuyt, 2005; Liu et al., 2016). Interestingly, in the most favorable microrefugium PmZS2a the frequency of *Z. sicula* trees is absolutely overwhelming within the woody plant community, suggesting a more effective competitiveness and space occupancy than in the two others refugia. In our case study, asexuality may eventually represent the winning strategy, and this may have enabled the survival of a peculiar genotype that proved to be successful in face of adverse environments, therefore assuring long‐term persistence of the current very small populations. On the other side, it is also recognized that strong isolation, perhaps lasting since millennia in the investigated area, promotes survival exclusively based on vegetative reproduction (Hampe & Petit, 2005; Kavecki, 2008; Mosblech et al., 2011).

Clonal plants are most often polyploid or issued from hybridization or both, and many evidences suggest that hybrid origin may be crucial for the success of asexual organisms in marginal habitats (Kavecki, 2008). As evoked in different studies (Christe et al., 2014; Denk & Grimm, 2005; Naciri et al., 2019), *Z. sicula* should issue from hybridization or even hybrid speciation at homoploid or polyploid level (Nieto Feliner, 2011). We guess that this process largely contributed to acquire specific adaptive traits involving a larger amount of genetic variation than the parental species, and above all, increasing the potential to adapt to novel habitats (Kavecki, 2008) such as those of current microrefugia. On the other hand, as suggested for *Quercus* species from Poland (Dzialuk et al., 2007) or *Lomatia tasmanica* from Tasmania (Lynch et al., 1998), triploidy most often associates with reduced fertility or even with absolute sexual sterility (Eckert, 2002). For the Sicilian relict populations, this trait is at odds with any possible natural migration toward other suitable sites (Carra et al., 2019), resulting in their definitive “entrapment” in the current isolated enclave locations.

## 5 CONCLUSIONS

Hydrological microrefugia play a crucial role in enabling the survival and preservation of mesic Cenozoic relict taxa, especially in arid environments. However, their functioning patterns can be manifold, depending on groundwater dynamics and patterns, thus investigating processes inherent to specific microrefugia is of paramount importance to understand the factors enabling the persistence of distinct relict taxa. The analyses focused on the topographic patterns of the last refugial areas of *Zelkova sicula* highlighted that convergent topographies support microhabitats that are significantly moister than the surrounding matrix, acting as effective microrefugia for this relict tree species. Such an assumption was confirmed by volumetric water content measurements, showing that in both locations, a conspicuous and vital water supply is still available to plants at the lowermost soil layers even during the driest months. Moreover, incised landforms, such as “Canyons/Deeply incised streams” and “U‐shaped valleys,” are assumed to be potentially more suitable in supporting higher water plant uptake, buffering more effectively seasonal fluctuations in precipitation. Accordingly, the prevailing occurrence of subpopulation PpZS2a in this type of convergent environments results in the significantly higher growth rate of local trees with respect to the two other population units.

However, some evidences (namely, recurrent decline due to severe drought stress, prevalent dwarf habit) suggest that the current microrefugia probably lie at the extreme boundary of the potential range of *Z. sicula*. According to this, they must be classified as “relative” hydrologic refugia that is microsites supporting refugia with a moisture availability different with respect to the surrounding landscape, but susceptible to experience critical shortage as a consequence of seasonality and regional climate instability. The marginality of such habitats and the long‐lasting strong isolation most likely involved clonality in *Z. sicula*, indeed a common feature in remnant plants species. Clonal growth, coupled to triploidy‐dependent sexual sterility, represents a major hurdle with respect to possible migration toward other suitable sites, condemning this Sicilian relict to its definitive confinement in the present enclave locations.

## CONFLICT OF INTEREST

The authors have no conflict of interest to declare.

## AUTHOR CONTRIBUTIONS

**Giuseppe Garfì:** Conceptualization (lead); data curation (lead); formal analysis (lead); funding acquisition (lead); investigation (lead); methodology (lead); project administration (equal); resources (lead); supervision (lead); writing – original draft (lead); writing – review and editing (lead). **Francesco Carimi:** Formal analysis (equal); project administration (equal); supervision (equal); writing – review and editing (equal). **Laurence Fazan:** Conceptualization (equal); investigation (equal); writing – review and editing (equal). **Alessandro Silvestre Gristina:** Data curation (equal); formal analysis (equal); methodology (equal); writing – review and editing (equal). **Gregor Kozlowski:** Conceptualization (equal); investigation (equal); writing – review and editing (equal). **Salvatore Livreri Console:** Data curation (equal); formal analysis (equal); methodology (equal); project administration (equal); writing – review and editing (equal). **Antonio Motisi:** Data curation (equal); writing – review and editing (equal). **Salvatore pasta:** conceptualization (equal); investigation (equal); writing – original draft (equal); writing – review and editing (equal).

## Data Availability

Data are archived in the publicly accessible repository Dryad. https://doi.org/10.5061/dryad.np5hqbzs1.

## APPENDIX 1

**TABLE A1:** List, surface and cover of the habitats reported in the Carta della Natura della Sicilia

**Habitat**	ZS1		ZS2	
	m^2^	*%*	m^2^	*%*
45.215 Southern Italian cork‐oak forests	410,997.9	*41*.*1*	300,797.0	*30*.*1*
41.732 Southern Italian and Sicilian *Quercus pubescens* woods	126,333.9	*12*.*6*	74,054.7	*7*.*4*
31.81 Medio‐European rich‐soil thickets (1)	200,921.5	*20*.*1*	292,230.4	*29*.*2*
33.6 Italian *Sarcopoterium spinosum* phryganas	59,412.0	*5*.*9*	200,582.7	*20*.*1*
32.215 *Calicotome* brush (2)	82,000.7	*8*.*2*	0.0	*0*.*0*
44.713 Sicilian Plane tree canyons (3)	13,192.3	*1*.*3*	0.0	*0*.*0*
34.633 Diss steppes (4)	106,854.0	*10*.*7*	0.0	*0*.*0*
34.36 Phoenician torgrass sward (5)	0.0	*0*.*0*	124,523.8	*12*.*5*
22.1 Fresh waters	287.7	*0*.*0*	0.0	*0*.*0*
83.11 Olive groves	0.0	*0*.*0*	4,276.2	*0*.*4*
82.3 Extensive cultivation	0.0	*0*.*0*	1,651.6	*0*.*2*
86.43 Railroad switch yards and other open spaces	0.0	*0*.*0*	1,883.7	*0*.*2*
Total	1,000,000.0	*100*.*0*	1,000,000.0	*100*.*0*

Note

Further explanations: (1) Shrublands dominated by woody plants mostly belonging to Rosaceae family, e.g. *Pyrus spinosa* Forssk., *Prunus spinosa* L. and *Rubus ulmifolius* Schott; (2) Calicotome = currently named *Cytisus infestus* C. Presl; (3) Plane tree = *Platanus orientalis* L.; (4) Diss = *Ampelodesmos mauritanicus* (Poir.) T. Durand et Schinz; (5) Phoenician torgrass = *Brachypodium retusum* (Pers.) P. Beauv.

scientific plants' name and surface % in italics.
